# Comparative proteomic analysis of virulent and avirulent strains of *Toxoplasma gondii* reveals strain-specific patterns

**DOI:** 10.18632/oncotarget.19077

**Published:** 2017-07-07

**Authors:** Dong-Hui Zhou, Ze-Xiang Wang, Chun-Xue Zhou, Shuai He, Hany M. Elsheikha, Xing-Quan Zhu

**Affiliations:** ^1^ State Key Laboratory of Veterinary Etiological Biology, Key Laboratory of Veterinary Parasitology of Gansu Province, Lanzhou Veterinary Research Institute, Chinese Academy of Agricultural Sciences, Lanzhou, Gansu Province, 730046, PR China; ^2^ Faculty of Medicine and Health Sciences, School of Veterinary Medicine and Science, University of Nottingham, Sutton Bonington Campus, Loughborough, LE12 5RD, UK; ^3^ Department of Parasitology, Shandong University School of Basic Medicine, Jinan, Shandong Province, 250012, PR China; ^4^ College of Animal Science and Technology, Anhui Agricultural University, Hefei, Anhui Province, 230036, PR China

**Keywords:** Toxoplasma gondii, oocyst, proteomics, iTRAQ, differentially expressed protein (DEP)

## Abstract

Research exploring the proteome of *Toxoplasma gondii* oocysts has gained momentum over the past few years. However, little is known about the oocyst's protein repertoires that contribute to differential virulence among *T. gondii* strains. Here, we used isobaric tag for relative and absolute quantitation-based proteomic analysis of oocysts of two *T. gondii* strains exhibiting the virulent PYS (ToxoDB#9) phenotype versus the less virulent PRU (Type II, ToxoDB#1) phenotype. Our aim was to determine protein expression patterns that contribute to the virulence of a particular phenotype. A total of 2,551 proteins were identified, of which 374 were differentially expressed proteins (DEPs) (|log_2_ fold change| ≥ 0.58 and *P* < 0.05). DEPs included 192 increased and 182 decreased proteins. Gene Ontology and KEGG pathway analyses revealed a large number of DEPs enriched in various metabolic processes. Protein interaction network analysis using STRING identified inosine monophosphate dehydrogenase (IMPDH), Bifunctional GMP synthase/glutamine amidotransferase protein, Glucose-6-phosphate 1-dehydrogenase, and Citrate synthase as the top four hubs. Of the 22 virulence proteins commonly expressed in the oocysts of the two strains, 13 and 2 proteins were increased in PYS strain and PRU strain, respectively. Also, 10 and 3 of the 22 identified oocyst wall proteins showed higher expression in oocysts of PRU strain and PYS strain, respectively. These findings revealed new proteomic differences in the oocysts of *T. gondii* strains of different genotypic backgrounds.

## INTRODUCTION

The apicomplexan protozoan parasite *Toxoplasma gondii* has a remarkable ability to infect a wide range of mammals, birds and humans [1–4]. Its indirect lifecycle involves asexual phase in the intermediate vertebrate host and sexual phase that occurs exclusively in the intestinal epithelium of the definitive felid host. *T. gondii* undergoes stage transformation during its development from tachyzoite to bradyzoite in the intermediate host to oocyst's stage in the feline definitive host. Infection can occur via ingestion of the cat-borne oocysts through contaminated food or water, ingestion of cysts in the tissues of an infected intermediate host or transplacentally from infected mothers to their offspring [5]. The ability of the different forms of the parasite's life cycle, in particular the oocyst stage, to adapt to and interact with various niches inside and outside the host is essential for successful colonization and infection [6]. Oocysts have an exceptional ability to adapt to adverse environmental conditions outside the host [7]. They can remain viable for at least 1 year at 4°C [8]. Oocysts remained viable for at least 4.5 years in fresh water and 2 years in marine water [9]. Exposure to ultraviolet irradiation at doses even exceeding those used to treat water did not fully inactivate *T. gondii* oocysts [10]. Sodium hypochlorite and ozone, two chemicals commonly used to treat sewage and drinking water, failed to inactivate all infective oocysts [11]. Interestingly, clinical illness can be more severe when hosts acquire the infection through ingestion of oocysts [12].

The clinical consequence of *T. gondii* infection in human ranges from asymptomatic to debilitating or even life threatening. There are likely many reasons for these differences. *T. gondii* displays a high degree of genetic diversity and includes strains with distinct virulence potentials. Previous studies identified three clonal lineages of *T. gondii* that exhibit different virulence phenotypes in mice, with type I strains are lethal in mice, but type II and III strains are less virulent [13–14]. In addition to the parasite genotype and virulence [13–16], the age and immune status of the infected host [4], and stage of the life cycle causing infection [12] were all found to influence the severity of toxoplasmosis. Also, the pathogenicity of *T. gondii* strains has been found to be correlated with their migratory capacity, the growth rate *in vitro* and the parasite loads in mice [15], and polymorphism and differential expression of the dense granule and rhoptry proteins [16]. Despite significant progress made in recent years in understanding the molecular basis of different pathogenic behaviors of distinct *T. gondii* genotypes, the mechanisms that underpin differences in the outcome of infection are not completely known.

In recent years, interests in *T. gondii* oocysts have increased due to their important role in the transmission of *T. gondii* to new hosts and ecosystems [17], and because of their links to increased infections in marine mammals and water-borne outbreaks in humans [18]. Transcriptomic and proteomic studies addressing the molecular structure of oocysts have been reported [19–22]. Proteins uniquely expressed in the oocysts have been identified [19–21] and proteins that mediate the adaptation of oocysts to nutrient-poor and stressing extracellular environment have been discovered [23]. Additionally, proteomic changes that occur during oocyst sporulation have been revealed [24–25]. However, knowledge of the differentially expressed proteins that promote virulence between strains of different genotypes is still limited. The availability of phenotypically distinct strains – virulent type I strain and less virulent type II strain of *T. gondii* – provides the opportunity for identifying strain-specific virulence factors. In the present study, we compared oocyst's protein expression of the virulent ToxoDB#9 (PYS) strain and the less virulent ToxoDB#1 (PRU) strain using iTRAQ (isobaric tag for relative and absolute quantitation)-based LC-MS/MS analysis. Our results revealed genotype-specific differences in the proteomes of oocysts of the two genotypic variants of *T. gondii* oocysts.

## RESULTS AND DISCUSSION

The aim of the study was to compare the proteomic profiles of virulent PYS strain with avirulent PRU *T. gondii* strain using quantitative iTRAQ-based proteomic and bioinformatic analyses. We tested whether there are phenotype-specific differentially expressed proteins that promote virulence and/or tolerance of the oocysts to environmental conditions. A total of 2,551 proteins were identified, of which 374 were differentially expressed proteins (DEPs) between the two parasite strains. Interestingly, about half of the GO terms under biological process and 12 pathways in the top 27 enriched KEGG pathways were related to metabolic activities, underscoring the roles of metabolism in the observed differences between the two parasite strains.

### Identification and expression of proteins

**Figure 1:**
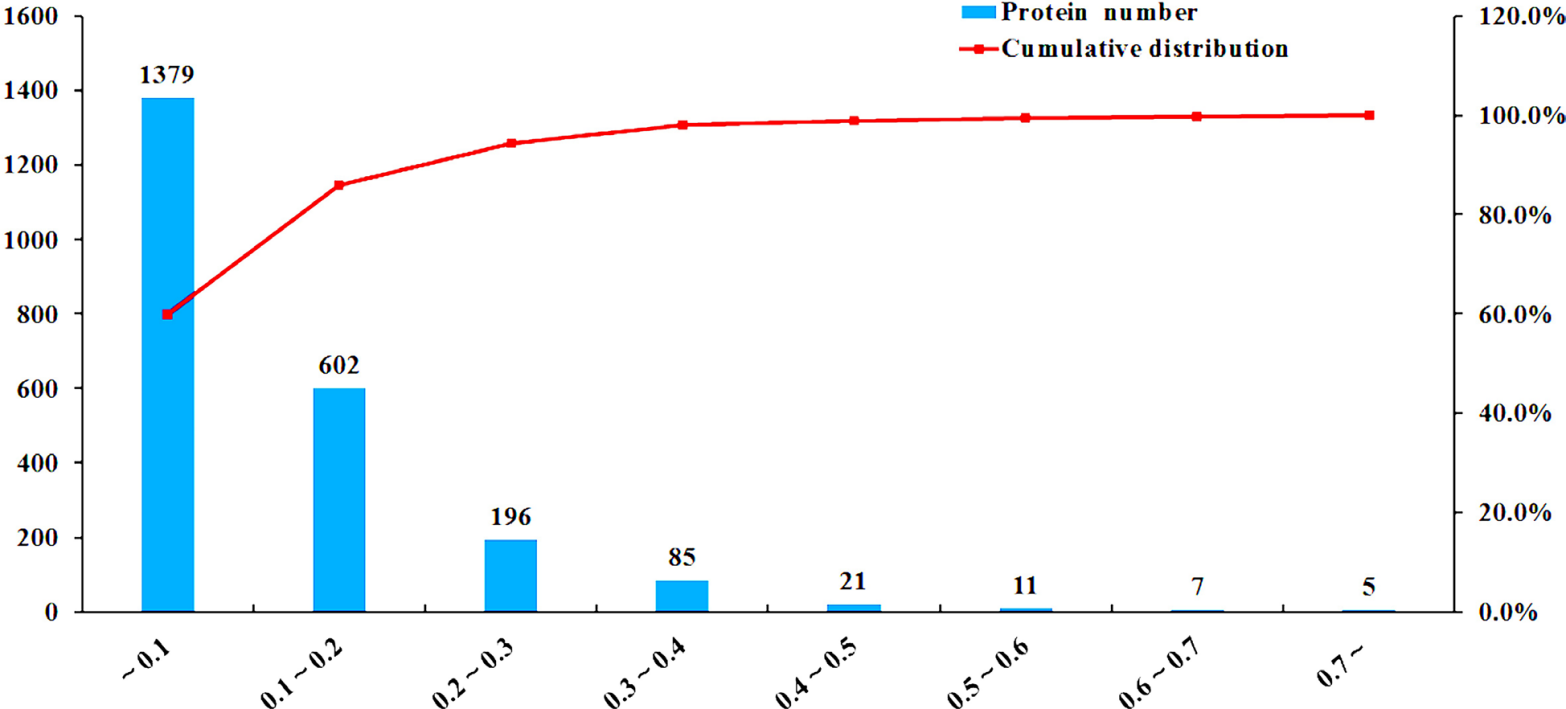
iTRAQ coupled with LC-MS/MS analysis of sporulated oocysts of ToxoDB#9 (PYS) strain and ToxoDB#1 (PRU) strain The x-axis represents coefficient of variation (CV) values. The left y-axis represents the number of proteins and the right y-axis represents the cumulative percentage of proteins (red lines).

**Figure 2:**
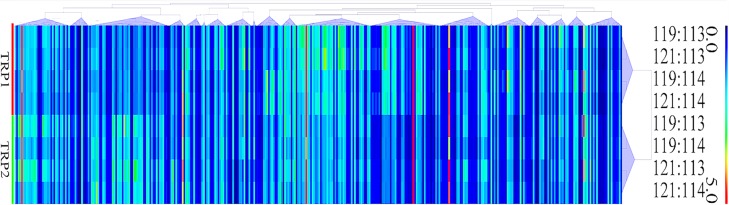
Hierarchical clustering of the relative abundance of oocyst's proteins of PYS strain versus PRU strain Each column represents one protein. Rows represent the pooled samples of the two technical replicates, TRP1 and TRP2. The expression levels of the proteins are shown in red (increase) and blue (decrease) colors.

**Figure 3:**
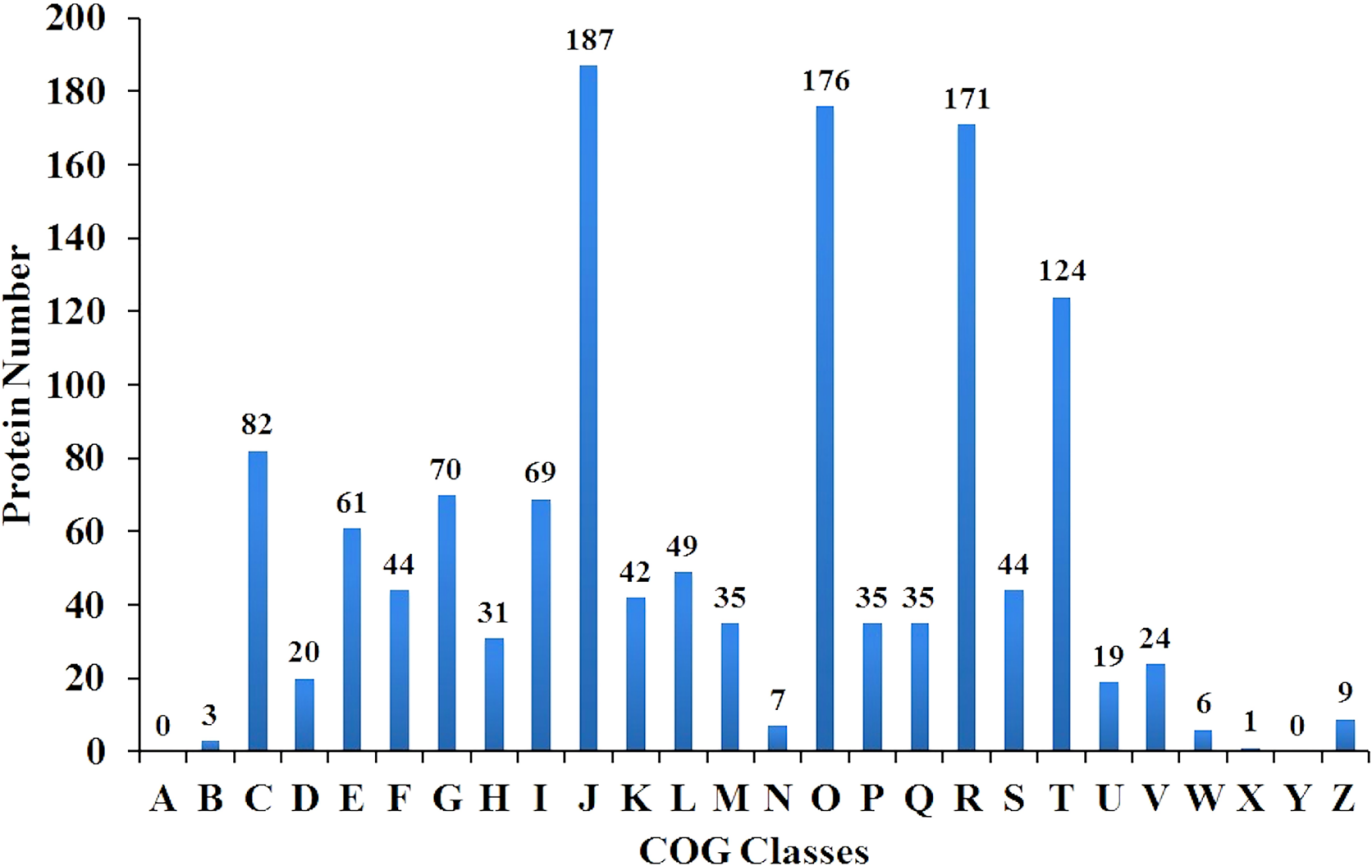
Distribution of cluster of orthologous groups (COG) analysis of the indentified proteins The y-axis represents the number of proteins. The x-axis represents different COG classes. A to Z represent: RNA processing and modification; Chromatin structure and dynamics; Energy production and conversion; Cell cycle control, cell division, chromosome partitioning; Amino acid transport and metabolism; Nucleotide transport and metabolism; Carbohydrate transport and metabolism; Coenzyme transport and metabolism; Lipid transport and metabolism; Translation, ribosomal structure and biogenesis; Transcription; Replication, recombination and repair; Cell wall/membrane/envelope biogenesis; Cell motility; Posttranslational modification, protein turnover, chaperones; Inorganic ion transport and metabolism; Secondary metabolites biosynthesis, transport and catabolism; General function prediction only; Function unknown; Signal transduction mechanisms; Intracellular trafficking, secretion, and vesicular transport; Defense mechanisms; Extracellular structures; Mobilome: prophages, transposons; Nuclear structure; Cytoskeleton, respectively.

A total of 404,046 spectra were obtained, of which 86,777 spectra were identified using MASCOT Server 2.4.0 (Matrix Science). Also, 51,728 distinct peptides were identified and 2,551 proteins were detected after grouping with unused score ≥ 1.3 and confidence value > 95%. The variation coefficients between the two biological replicates covered 99% of the identified proteins when the coefficient of variation (CV) < 50% (Figure 1). The results of hierarchical clustering analysis of the two biological replicates (each contained two pooled technical replicates) are shown in Figure 2. Cluster of orthologous group (COG) analysis divided the identified proteins into 26 categories. As shown in Figure 3, translation, ribosomal structure and biogenesis; posttranslational modification, protein turnover and chaperones; general function prediction only; signal transduction mechanisms; and energy production and conversion were the five categories with the most identified proteins.

DEPs were identified by comparing the relative protein expression values between PYS strain and PRU strain. A total of 374 proteins were classified as DEPs (|log_2_ fold change| ≥ 0.58 and *P* < 0.05), of which 192 were increased and 182 were decreased (Supplementary Table 1 and Supplementary Table 2). GTP-binding protein lepA (TGME49_307980), *Toxoplasma gondii* family D protein (TGME49_271590), TGME49_294600, TGME49_205090, and toxofilin (TGME49_214080) were the top five increased proteins. By contrast, the top five mostly decreased proteins were dense granule protein GRA7 (TGME49_203310), TGME49_203890, rhoptry protein ROP5 (TGME49_308090), Zn-containing alcohol dehydrogenase (TGME49_311780), and nudix-type motif 9 isoform a family protein (TGME49_247220).

### Functional annotation of DEPs

**Figure 4:**
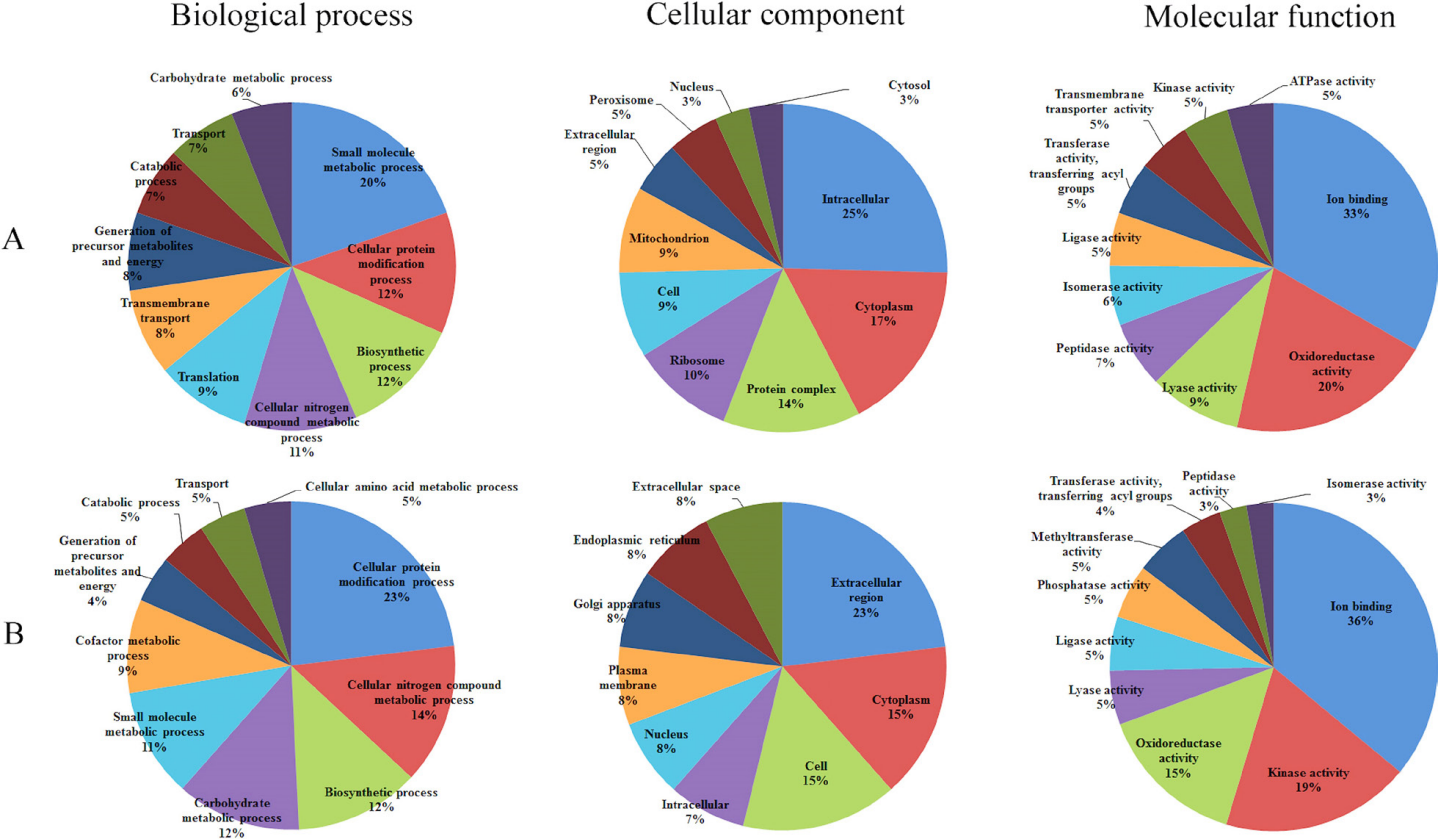
Gene Ontology (GO) distribution of the differentially expressed proteins (DEPs) The top 10 enriched GO terms under Biological Process, Cellular Component and Molecular Function for (**A**) increased proteins and (**B**) decreased proteins.

To elucidate the biological roles of the DEPs between oocysts of PYS and PRU strains, GO enrichment analysis using GOseq R package was performed. The differentially increased and decreased proteins were classified into three GO categories, namely biological process, cellular component and molecular function. The top 10 most abundant GO terms in the three categories are presented for increased and decreased proteins (Figure 4A–4B). The top five enriched GO terms for increased proteins under biological process included small molecule metabolic process, cellular protein modification process, biosynthetic process, cellular nitrogen compound metabolic process, and translation. By contrast, cellular protein modification process, cellular nitrogen compound metabolic process, biosynthetic process, carbohydrate metabolic process, and small molecule metabolic process were the top five abundantly enriched terms for the decreased proteins.

**Figure 5:**
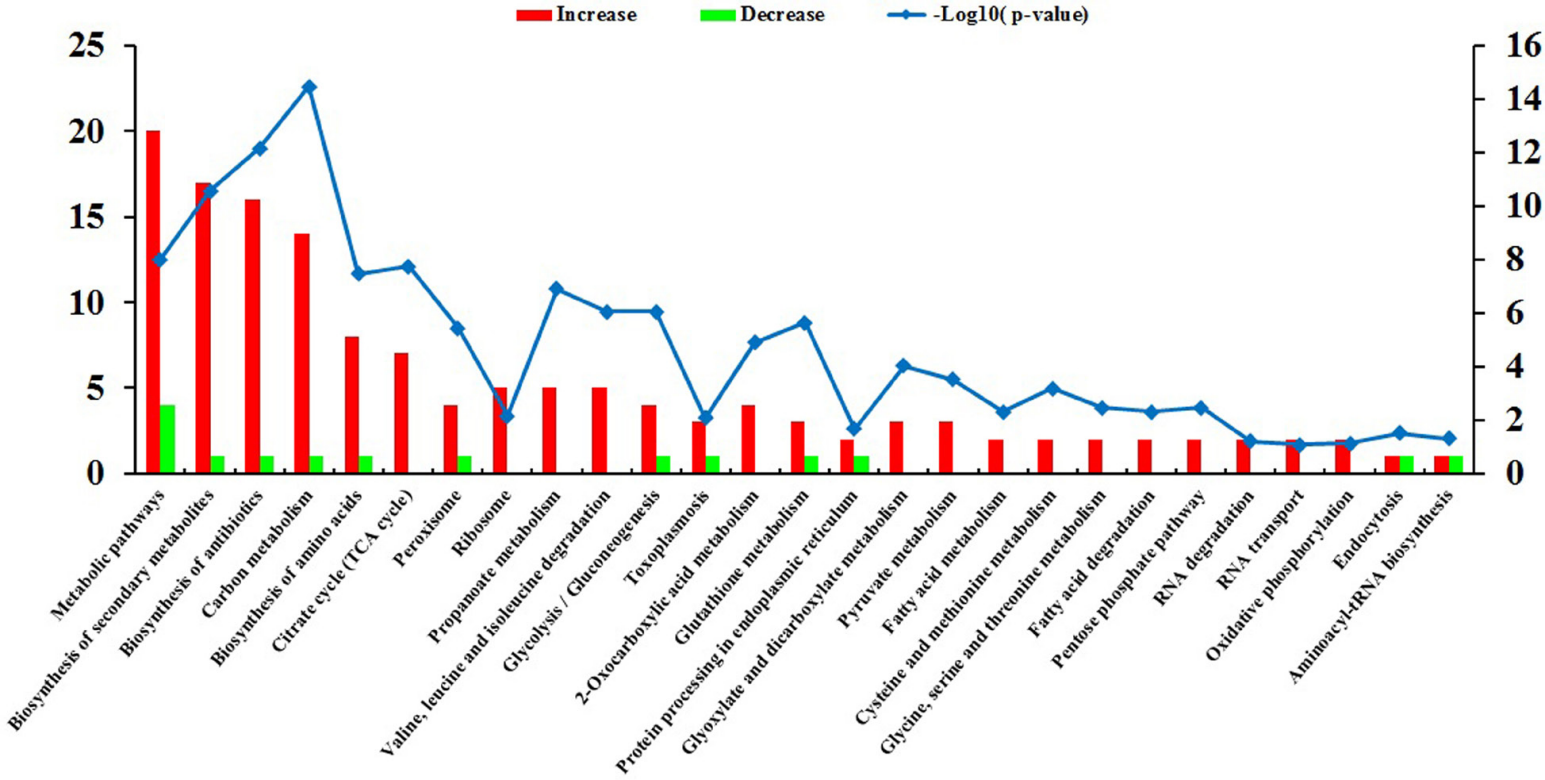
KEGG pathway analysis of the differentially expressed proteins (DEPs) The horizontal axis represents the significantly enriched KEGG pathways. The left y-axis represents the number of DEPs and the right y-axis represents logarithmic corrected *p*-values for significant overrepresentation [–log10(*p*–value)].

The top three increased GO terms of the cellular component category included intracellular, cytoplasm and protein complex, whereas the top three decreased terms included extracellular region, cytoplasm and cell. In the category of molecular function, ion binding was the most significantly enriched GO term. KEGG pathway enrichment analysis revealed 164 DEPs, which had a KEGG Orthology (KO) ID and were related to 51 pathways. The 27 highly enriched pathways are represented in Figure 5. The 10 most significantly enriched of which were metabolic pathways, biosynthesis of secondary metabolites, biosynthesis of antibiotics, carbon metabolism, biosynthesis of amino acids, Citrate cycle (TCA cycle), peroxisome, ribosome, propanoate metabolism, and valine, leucine and isoleucine degradation.

### Differences in the expression of virulence factors

**Figure 6:**
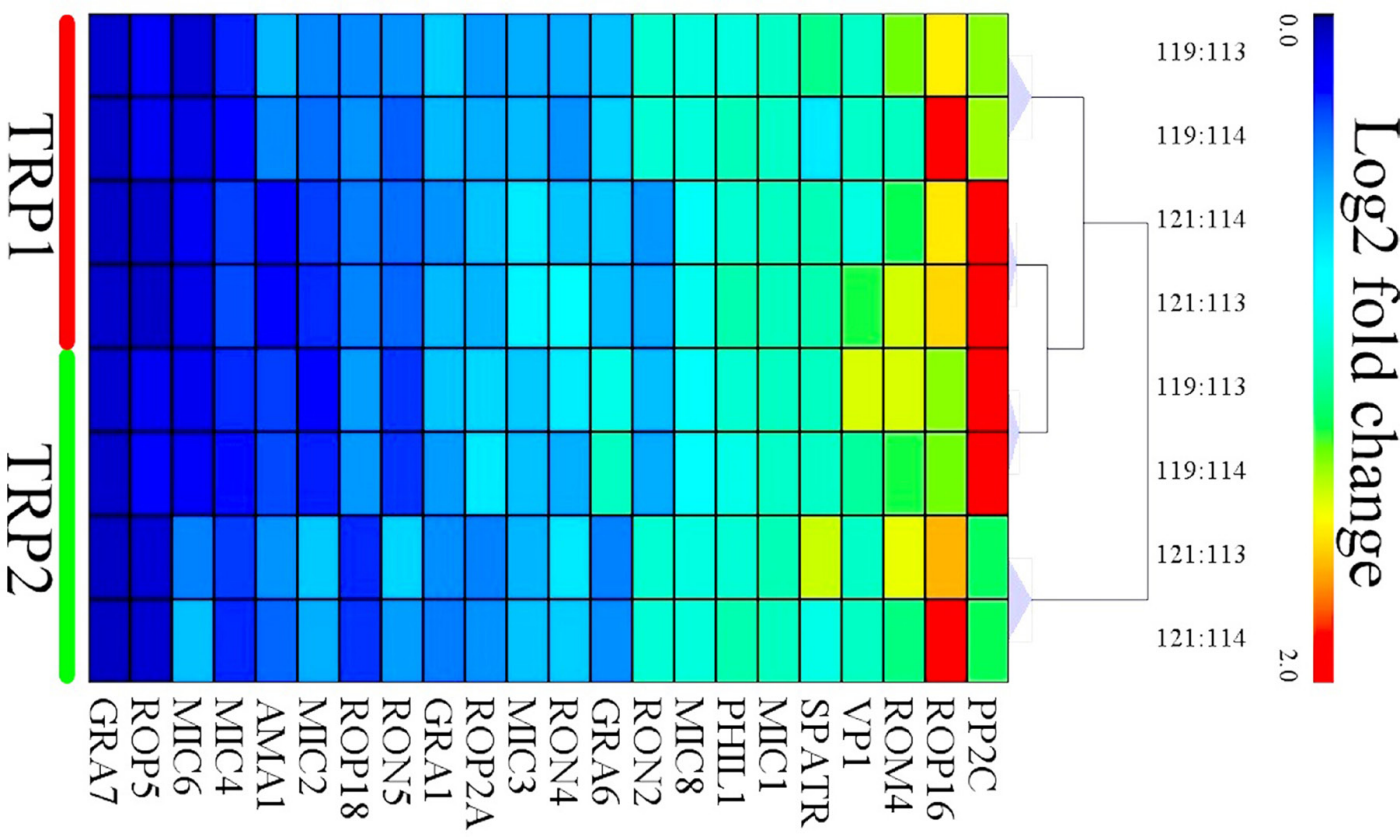
Heat map clustering of the intensities of the differentially expressed (DE) virulence proteins of oocysts between PYS strain and PRU strain Expression values were log_2_-transformed and were annotated based on a gradient color scheme. The x-axis represents the logogram of virulence proteins. The TRP1 and TRP2 in y-axis represent technical replicates 1 and 2, respectively.

A number of virulence factors of *T. gondii* oocysts have been shown to be increased during sporulation and these have been linked to the infectivity of sporulated oocysts [25]. In our study, we detected differences in the expression levels of these virulence proteins between sporulated oocysts of PYS and PRU strains. Among the 22 virulence proteins commonly expressed in the sporulated oocysts of PYS strain and PRU strain, only serine-threonine phosphatase 2C (PP2C) and rhoptry protein ROP16 (ROP16) had a higher expression level in the PRU strain. By contrast, 13 virulence factors, including dense granule protein GRA6 (GRA6), rhoptry neck protein RON4 (RON4), microneme protein MIC3 (MIC3), rhoptry protein ROP2A (ROP2A), dense granule protein GRA1 (GRA1), rhoptry neck protein RON5 (RON5), rhoptry protein ROP18 (ROP18), microneme protein MIC2 (MIC2), apical membrane antigen AMA1, microneme protein MIC4 (MIC4), microneme protein MIC6 (MIC6), rhoptry protein ROP5 (ROP5), and dense granule protein GRA7 (GRA7) were upregulated in the PYS strain. Furthermore, the level of expression of the following seven virulence proteins did not show any significant difference between the two strains: rhomboid protease ROM4 (ROM4), V-type H(+)-translocating pyrophosphatase (VP1), sporozoite protein with an altered thrombospondin repeat (SPATR), microneme protein MIC1 (MIC1), photosensitized INA-labeled protein PHIL1 (PHIL1), microneme protein MIC8 (MIC8), and rhoptry neck protein RON2 (RON2) (Figure 6). These results indicate that sporulated oocysts of the virulent PYS strain express more virulence factors than sporulated oocysts of the less virulent PRU strain.

### Expression of oocyst wall proteins

Oocyst wall proteins play important roles in the environmental tolerance of *T. gondii* oocysts and can protect the sporozoites from adverse conditions outside in the environment and inside the host [24]. The protein profile of *T. gondii* oocyst wall has already been identified [27–28]. However, in the present study we identified 22 oocyst wall proteins and analyzed their differential expression between two distinct genotypic variants of *T. gondii* oocysts. As shown in Table 1, 10 oocyst wall proteins were increased and three were decreased. Roughly half of the oocyst wall proteins had higher expression level in PRU oocysts compared with PYS oocysts. Only three oocyst wall proteins were overexpressed in PYS oocysts compared with PRU oocysts. The other nine oocyst proteins showed no significant difference between the two parasite strains. These discrepancies in the expression of oocyst wall proteins are likely related to differences in the ability of oocysts of PYS strain and PRU strain to survive in the environment.

**Table 1:** Differential expression of oocyst wall proteins of *Toxoplasma gondii*

Gene ID	Protein Description	CV	AVE	Differential Expression
TGME49_235315	PAN domain-containing protein	0.0645	2.4260	Up
TGME49_209610	oocyst wall protein OWP2 (OWP2)	0.1685	2.4252	Up
TGME49_286250	hypothetical protein	0.1774	2.2303	Up
TGME49_232170	hypothetical protein	0.1143	2.2166	Up
TGME49_235390	PAN domain-containing protein	0.0756	1.8167	Up
TGME49_209470	hypothetical protein	0.0584	1.7690	Up
TGME49_320530	hypothetical protein	0.3891	1.6981	Up
TGME49_281590	hypothetical protein	0.1065	1.6710	Up
TGME49_253150	hypothetical protein	0.1386	1.5380	Up
TGME49_294820	type I fatty acid synthase	0.1292	1.5107	Up
TGME49_316550	hypothetical protein	0.1300	1.2186	N
TGME49_268310	oocyst wall protein OWP3 (OWP3)	0.0640	1.1597	N
TGME49_269380	hypothetical protein	0.1047	1.1517	N
TGME49_248810	nuclear factor NF7	0.1110	1.1005	N
TGME49_254430	microneme protein	0.0239	0.9752	N
TGME49_204420	oocyst wall protein OWP1 (OWP1)	0.0374	0.9513	N
TGME49_203500	Alanine dehydrogenase/pyridine nucleotide transhydrogenase domain-containing protein	0.1033	0.9247	N
TGME49_237080	hypothetical protein	0.2250	0.8431	N
TGME49_258910	hypothetical protein	0.1331	0.5933	Down
TGME49_319890	hypothetical protein	0.2363	0.5931	Down
TGME49_306050	hypothetical protein	0.1883	0.5412	Down
TGME49_287250	hypothetical protein	0.5441	0.5079	N

Abbreviations: CV: coefficient of variation; AVE: average expression value of two biological replicates; Up: increased; Down: decreased; N: non-significant change.

### Protein-protein network construction

**Figure 7:**
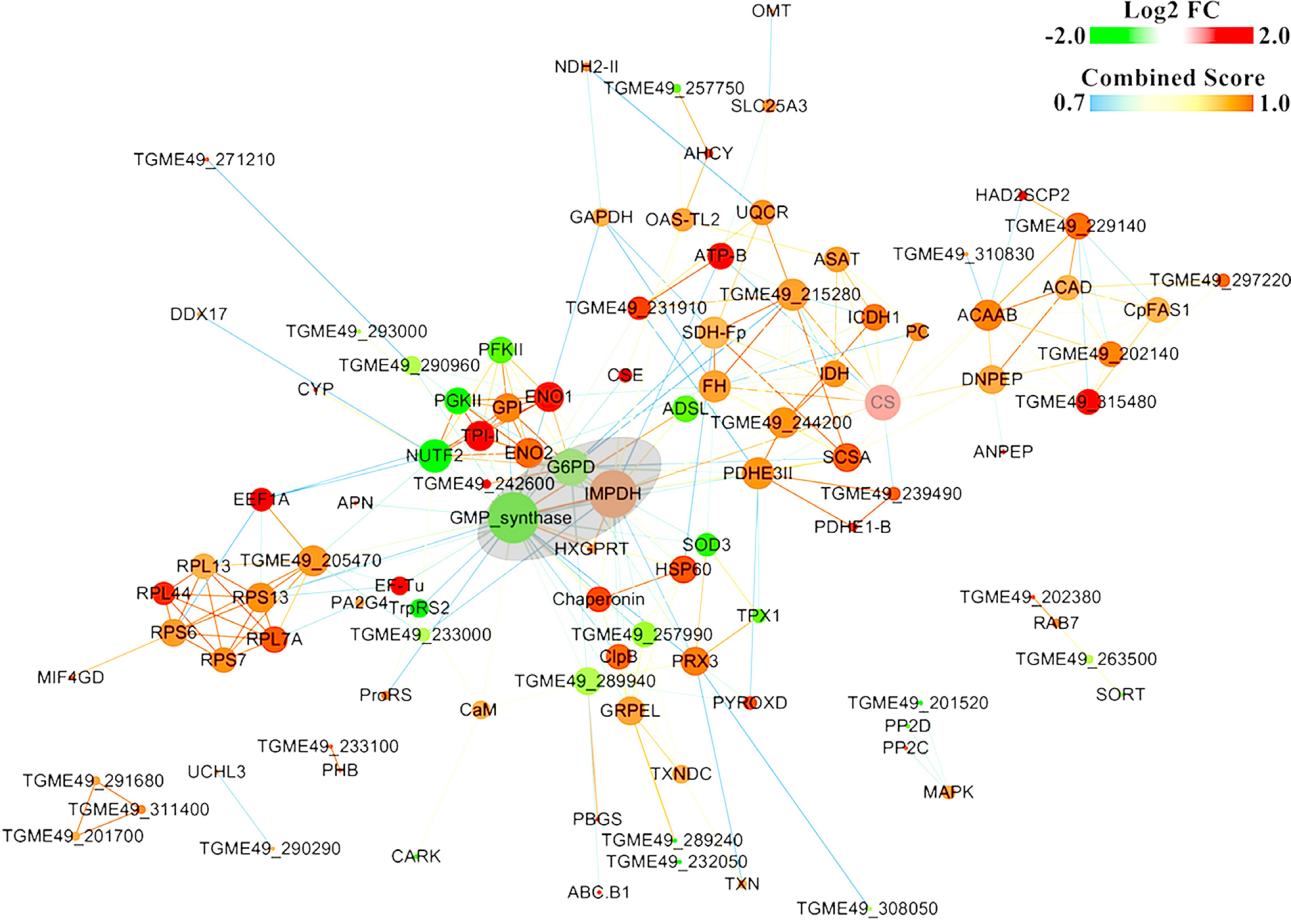
Constructed protein-protein interaction (PPI) networks of the DEPs PPI networks of the differentially expressed proteins (DEPs) were predicted using STRING and were illustrated using Cytoscape software. Nodes represent DEPs and edges represent interactions between two proteins. Red- and green-colored nodes represent products of increased and decreased DEPs, respectively. The node size indicates high (large) or low (small) degree of interaction. Proteins that are associated to each other are linked by an edge. The color of the edge indicates the combined interaction score (edge score).

**Figure 8:**
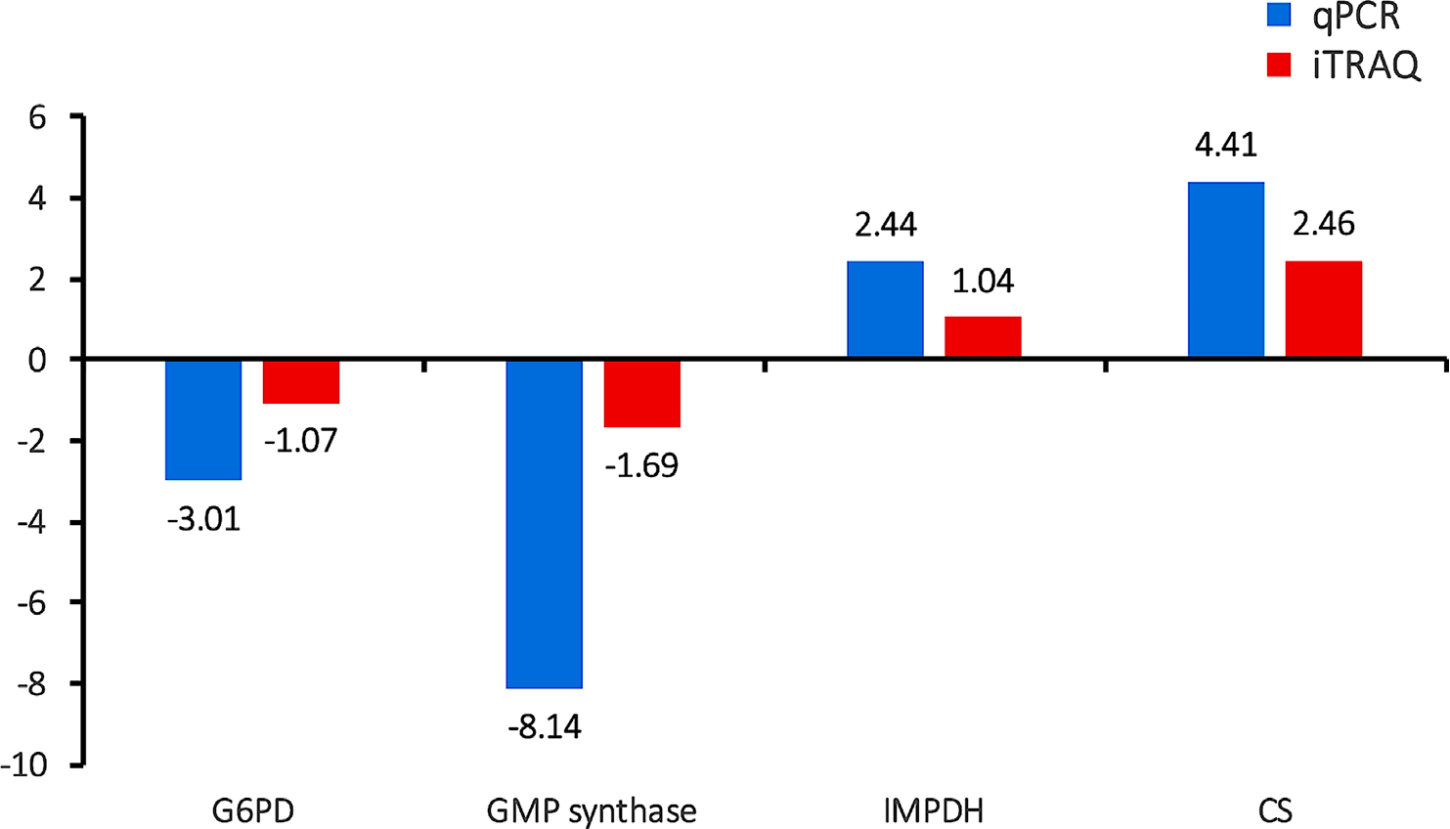
Validation of the relative abundance of the top four hub DEPs by qPCR x-axis shows different DEPs and y-axis shows log_1.5_ fold change values.

The protein-protein interaction (PPI) (combined score > 0.9) networks of the DEPs were built using Cytoscape software (Figure 7). The interaction network included 102 nodes and 271 edges. The degrees of interaction were defined to determine the number of neighbors that a node is directly linked to, and nodes with a high degree of interaction with other proteins were defined as ‘hub’ proteins. The significant hub proteins were inosine monophosphate dehydrogenase (IMPDH), Bifunctional GMP synthase/glutamine amidotransferase protein (GMP synthase), Glucose-6-phosphate 1-dehydrogenase (G6PD), and Citrate synthase (CS). All these protein hubs are known to play important roles in various metabolic processes. For example, IMPDH and GMP synthase are key enzymes in the synthesis of guanine nucleotides and have been considered good therapeutic targets [29–30]. Likewise, G6PD and the mitochondrial CS play a role in glycolysis and carbohydrate metabolism. The results of qPCR validation of these four hub proteins were consistent with the proteomic data (Figure 8). The expression level of G6PD and GMP were lower and the expression level of IMPDH and CS were higher in the PYS strain compared to the PRU strain.

In conclusion, we employed iTRAQ-based proteomic approach to profile the proteomic differences in *T. gondii* oocysts between the virulent PYS strain and the less virulent PRU strain. A total of 2,551 proteins were identified, of which 374 were differentially expressed. KEGG pathway enrichment analysis revealed that the most enriched pathways were related to metabolism and bioenergetics. While PYS oocysts showed significant increase in the expression of virulence-related proteins compared to PRU oocysts, oocyst wall proteins were highly expressed in PRU oocysts compared with PYS oocysts. Further research is needed to determine how these differentially expressed proteins contribute to the virulence of PYS strain and the environmental endurance of PRU strain. These findings contribute to our understanding of the virulence determinants of *T. gondii* strains of different genotypes and may facilitate the identification of new therapeutic targets for prevention and control of toxoplasmosis.

## MATERIALS AND METHODS

### Ethics statement

All animal protocols were reviewed and approved by the Animal Administration and Ethics Committee of Lanzhou Veterinary Research Institute, Chinese Academy of Agricultural Sciences. Animals were purchased from Laboratory Animal Center of Lanzhou Veterinary Research Institute. The study was performed in strict accordance with the recommendations set forth in the Animal Ethics Procedures and Guidelines of the People's Republic of China. All efforts were made to minimize animal suffering and to reduce the number of animals used to produce *T. gondii* parasites.

### Production and purification of sporulated oocysts

The virulent PYS (ToxoDB#9 genotype) and less virulent Prugniuad (PRU; ToxoDB #1) strains were used in the present study because ToxoDB#9 (Chinese 1) is the predominant *T. gondii* genotype in Asia and *T. gondii* type II is the most prevalent genotype in cats. Both strains are routinely maintained in our laboratory. Briefly, 16-week-old female guinea pigs were intraperitoneally inoculated with 10^5^ tachyzoites of PYS strain. One month after the inoculation, guinea pigs showed lethargy, loss of appetite, panting, and diarrhea. Then, animals were euthanized and the cysts in their brain and muscle tissues were collected and used to infect a specific-pathogen-free kitten. Feces from the infected kitten was collected daily and examined by zinc sulfate double centrifugation to detect the excreted oocysts. The kitten was tested by indirect fluorescent-antibody test prior to infection to ensure that they were seronegative for *T. gondii* antibodies. The PRU strain was maintained via oral passage of cysts in six-week-old, female BALB/c mice. One week postinfection, infected mice exhibited reduced appetite, ruffled fur and head tilting. Specific-pathogen-free, 10-week-old, kitten was orally inoculated with 200 cysts and its feces was collected daily and examined as described above to detect oocysts of the PRU strain.

Oocysts of PYS and PRU strains were isolated from the cat feces by filtering fecal suspension through a tea strainer (250-μm pore size). Then, the fecal filtrates were pelleted by centrifugation at 60 g for 15 min. The supernatant was discarded and the pellet was washed 3× with phosphate-buffered saline (PBS). After discarding the supernatant, the pellet was suspended in sucrose solution to concentrate the oocysts. Following sucrose flotation, a cesium chloride (CsCl) density gradient method was used to purify *T. gondii* oocysts as described previously [26]. To induce sporulation, the purified oocysts were mixed with 2% H_2_SO_4_ and were aerated on a shaker at ambient temperature for 7 days. Then, sporulated oocyst were washed twice with 0.85% saline, mixed with 2% H_2_SO_4_ and stored at 4°C until analysis. Prior to use, oocysts were washed 3× in PBS to remove sulfuric acid and restore neutral pH.

### Protein extraction and iTRAQ labeling

Total protein was extracted from an equal number (∼10^7^) of sporulated oocysts of PYS and PRU strains. Following centrifugation at 350 g for 15 min, oocyst's pellets were suspended in a radioimmunoprecipitation assay (RIPA; 50 mM Tris-HCl, 150 mM NaCl, 1% SDS, 0.1% Trionx-100 and 1% SDC pH 8) buffer supplemented with phenylmethylsulfonyl fluoride (PMSF). The oocyst's suspensions were sonicated on ice and pelleted at 10,625 g for 20 min at 4°C. The supernatant was collected and the protein concentration was measured by BCA assay (QuantiPro^TM^ BCA Assay Kit, Sigma). About 100 μg protein of each sample was reduced, alkylated, and then precipitated by the methanol/chloroform precipitation method. Firstly, 4 μL of the Reducing Reagent were added into each sample followed by incubation at 60°C for 1 hr. Then, the sample was mixed with 2 μL Cysteine-Blocking Reagent and kept at ambient temperature for 10 min. Following rinsing of the 10KD millipore with 70% ethanol and deionized water, the protein solution was poured into millipore and centrifuged at 13,400 g for 20 min. Solution at the bottom of millipore was discarded and the millipore was centrifuged with 100 μL 0.25 M TEAB (tetraethylammonium bicarbonate) 3× at 13,400 g for 20 min. The protein pellets were reconstituted in 50 μL of 6M urea/50 mM TEAB with sonication and digested in 2% trypsin overnight (Promega). Then, peptides were labeled with 8-plex iTRAQ reagents (113, 114, 119, 121) according to the manufacturer's instructions (AB Sciex, USA). About 150 μL isopropyl alcohol were added into labeling reagent, and the mixture was added into the samples and incubated for 2 hr at ambient temperature. After termination with deionized water and centrifugation, samples were dried and maintained at −80°C until use.

### Strong cation exchange (SCX) fractionation and LC-MS/MS analysis

The iTRAQ labeled peptides were mixed in equal amounts and fractioned by a high performance liquid chromatography (HPLC) system using Phenomenex columns (Gemini-NX 3u C18 110A; 150*2.00 mm). The dried peptides were dissolved in 100 μL buffer A (20 mM NH4HCO3 in 3% acetonitrile, pH 10), thoroughly mixed and were then loaded onto Ultremex SCX column. Fractions were collected at a flow rate of 1mL/min with buffer B (20 mM NH_4_HCO_3_, pH 10) at gradients of 0–8% for 5 min; 8–45% for 25 min; 45–80% for 15 min; and 80–100% for 5 min. Twenty-four fractions were collected and acidified with 50% Trifluoroacetic acid (TFA). The peptide fractions were dried using vacuum centrifugation and reconstituted in 0.1% formic acid and 2% acetonitrile. Then, peptides were centrifuged at 13,447 g for 20 min and supernatant was transferred into analytical columns to be identified with an online Q Exactive system (Thermal Scientific). The first grade MS parameters included scan range 350 to1800 m/z with a resolution set at 70,000 with a maximum injection time 40 ms. The second grade MS spectra were acquired in a resolution of 17,500 with 60 ms maximum injection time and the 20 top precursors for each MS cycle were selected.

### Protein quantitation

Mascot generic format (.mgf) data file transformed from raw MS data by Proteome Discoverer™ 1.4 were queried to *T. gondii* ME49 strain database (http://www.ToxoDB.org/common/downloads/release-10.0/TgondiiME49/fasta/data/), which contains 8,322 protein sequences. Proteins were further identified and quantified with ProteinPilot^TM^ Software 4.5 (AB SCIEX). The parameters for identification and quantification were set as follows: FDR < 0.01 for identification of peptides and proteins; confidence level of 95% or unused confidence score larger > 1.3 for quantification. Proteins with fold change ratios ≥ 1.5 or ≤ 0.67 were designated as increased or decreased proteins, respectively.

### Bioinformatics analysis

To gain insight into the biological functions of DEPs, gene ontology (GO) classification and enrichment analyses (http://www.geneontology.org) were performed. GO provides a framework for functional annotation and classification of the protein expression data. DEPs were categorized into three main groups: molecular function, biological process and cellular component. Additionally, Kyoto Encyclopedia of Genes and Genomes (KEGG; http://www.genome.jp/kegg/) pathway enrichment analysis was performed to map the potential pathways of the DEPs. Hierarchical clustering analysis and presentation was performed with Cluster 3.0 and java Tree view, respectively. The detection of key protein-interacting ions in the PPI networks was important for the identification of the mechanisms and factors that underpin the virulence of *T. gondii* oocysts. We utilized the Search Tool for the Retrieval of Interacting Genes/Proteins (http://www.string-db.org/), a database of known and predicted protein interactions, to construct the PPI network and then visualized the distribution characteristics of increased and decreased DEPs in the network with Cytoscape software, version 3.2.0.

### Quantitative real-time PCR (q-PCR) validation

Q-PCR was employed to confirm the gene expression of the identified four hub proteins. TRIzol method was used to extract total RNA from sporulated oocysts of the PYS and PRU strains, respectively, according to the manufacture's instruction (Invitrogen). RNA templates were reverse transcribed to cDNA using a reverse transcription kit (Promega). The *T. gondii* β-actin was chosen as an endogenous reference gene to normalize all q-PCR data. Q-PCR reactions were performed on the Rotor-Gene Q (QIAGEN) with SYBR Green GoTaq^®^ qPCR Master Mix (Promega). Primers used in this study are shown in Table 2. The conditions performed in q-PCR cycle were as follows: 95°C for 2 min followed by 40 cycles of 95°C for 15 s, 60°C for 30 s, 72°C for 30 s. The 2^−ΔΔCT^ method was applied to calculate the relative gene expression [31].

**Table 2:** Genes and primers used in qPCR validation

Gene	Primer name	Primer sequence (5′ to 3′)
β-actin	β-actin-F	CGAGAGGCTGACCAAGGAAC
	β-actin-R	CTGCTGGAAGGTGGAGAGAGA
G6PD	G6PD-F	GACGCCTCTGCCGATTTACT
	G6PD-R	TCGCTTCCCTTCGCATCT
GMP synthase	GMP synthase-F	GGACTTTATGACGGCTGACTGG
	GMP synthase-R	TGATGCCCTTGACTTCGTTG
IMPDH	IMPDH-F	GAAACTCTGCCTCCTCCTCTC
	IMPDH-R	AAGACACCCACAAGCCAAAC
CS	CS-F	CCGCAACGCTTAGGAGACTA
	CS-R	TACAGGGATTGGCTTCTGGA

### Data and materials availability

All the mass spectrometry data have been submitted to the ProteomeXchange Consortium with identifier PXD005928.

## SUPPLEMENTARY MATERIALS TABLES






