# Supplementary material for: Comparative proteomic analysis of virulent and avirulent strains of Toxoplasma gondii reveals strain-specific patterns
Source: Oncotarget. 2017 Jul 7;8(46):80481–91. doi: 10.18632/oncotarget.19077 (PMC5655214; doi:10.18632/oncotarget.19077)
Supplement: Supplementary file 1 [file oncotarget-08-80481-s001.pdf]

# Comparative proteomic analysis of virulent and avirulent strains of *Toxoplasma gondii* reveals strain-specific patterns

## SUPPLEMENTARY MATERIALS

**Supplementary Table 1: List of increased proteins of the sporulated oocysts between ToxoDB#1 (PRU) strain and ToxoDB#9 (PYS) strain (|log2 fold change| > 0.58 and $P < 0.05$).** See Supplementary_Table_1

**Supplementary Table 2: List of decreased proteins of the sporulated oocysts between ToxoDB#1 (PRU) strain and ToxoDB#9 (PYS) strain (|log2 fold change| > 0.58 and $P < 0.05$).** See Supplementary_Table_2
